# A randomised trial of robotic and open prostatectomy in men with localised prostate cancer

**DOI:** 10.1186/1471-2407-12-189

**Published:** 2012-05-25

**Authors:** Robert A Gardiner, John Yaxley, Geoff Coughlin, Nigel Dunglison, Stefano Occhipinti, Sandra Younie, Rob Carter, Scott Williams, Robyn J Medcraft, Nigel Bennett, Martin F Lavin, Suzanne Kathleen Chambers

**Affiliations:** 1 University of Queensland Centre for Clinical Research, Royal Brisbane Hospital, Queensland, Australia; 2 Department of Urology, Royal Brisbane Hospital, Queensland, Australia; 3 Edith Cowan University Health and Wellness Institute, Edith Cowan University, Western Australia, Australia; 4 Griffith Health Institute, Griffith University, Queensland Victoria,, Australia; 5 Deakin Health Economics, Deakin University, Victoria, Australia; 6 Peter MacCallum Cancer Centre, Queensland, Australia; 7 Radiation Biology and Oncology Laboratory, Queensland Institute of Medical Research, Victoria, Australia; 8 Cancer Council Queensland, Queensland, Australia

## Abstract

**Background:**

Prostate cancer is the most common male cancer in the Western world however there is ongoing debate about the optimal treatment strategy for localised disease. While surgery remains the most commonly received treatment for localised disease in Australia more recently a robotic approach has emerged as an alternative to open and laparoscopic surgery. However, high level data is not yet available to support this as a superior approach or to guide treatment decision making between the alternatives. This paper presents the design of a randomised trial of Robotic and Open Prostatectomy for men newly diagnosed with localised prostate cancer that seeks to answer this question.

**Methods/design:**

200 men per treatment arm (400 men in total) are being recruited after diagnosis and before treatment through a major public hospital outpatient clinic and randomised to 1) Robotic Prostatectomy or 2) Open Prostatectomy. All robotic prostatectomies are being performed by one surgeon and all open prostatectomies are being performed by one other surgeon. Outcomes are being measured pre-operatively and at 6 weeks and 3, 6, 12 and 24 months post-surgery. Oncological outcomes are being related to positive surgical margins, biochemical recurrence +/− the need for further treatment. Non-oncological outcome measures include: pain, physical and mental functioning, fatigue, summary (preference-based utility scores) and domain-specific QoL (urinary incontinence, bowel function and erectile function), cancer specific distress, psychological distress, decision-related distress and time to return to usual activities. Cost modelling of each approach, as well as full economic appraisal, is also being undertaken.

**Discussion:**

The study will provide recommendations about the relative benefits of Robotic and Open Prostatectomy to support informed patient decision making about treatment for localised prostate cancer; and to assist in treatment services planning for this patient group.

**Trial registration:**

ACTRN12611000661976

## Background

Because of an absence of objective information from randomised studies, it is advised that men diagnosed with localised prostate cancer (PCa) are offered three broad treatment approaches that include surgery or radical prostatectomy (RP), irradiation of the prostate, or observation in the form of active surveillance or watchful waiting [1]. In practice, however, multiple options are often presented that arise not only through treating clinicians, but from family, friends, the media, and self-help programs [2]. The option list can then include an array such as open, laparoscopic & robotic RP, external beam radiation, high and low-dose brachytherapy, high frequency focussed ultrasound, a variety of alternative therapies and monitoring. For each approach, the newly diagnosed man must consider costs and benefits from a survival and quality of life (QoL) point of view, financial costs and relative availabilities.

In this decision context, high quality evidence about the expected outcomes of each approach is essential however, problematic for robotic procedures, this information is lacking. Specifically, data on QoL outcomes comparing robotic procedures with open RP are typically from case series from single centres [3-6], with, to our knowledge, only one other randomised controlled trial being undertaken, comparing open, laparoscopic and robotic prostatectomy (the LopeRA study) which is experiencing difficulty recruiting patients [7,8]. This paucity of high-quality information leaves men with localised prostate cancer, and their physicians, in the difficult position of trying to make a treatment decision without high quality evidence. Moreover there is evidence that once robotic surgery becomes available, consumer demand drives up its usage, with one study reporting that during the course of a case series to compare robotics with other approaches, at the study completion more than 94% of men had chosen robotic surgery [5]. This means there are also health services planning and delivery issues that arise from this evidence gap.

To date, case series suggest varied outcomes when comparing robotic with open RP. Some suggest that patients who undergo robotic surgery have less post-operative narcotic use, shorter hospital stays [6] and better short term urinary and sexual function [3,5]. Other studies have found no clear differences in functional outcomes with regards to potency, continence [9], pain and recovery [10]. Finally, the methodological issues that ensue from selection bias in case series and the use of non-standard reporting methods make much of this data difficult to interpret [4,11].

The present study will address this knowledge gap by completing the first randomised trial with men with localised prostate cancer comparing robotic with open RP and assessing QoL and psychosocial outcomes using reliable and previously validated measures of pain, physical and mental functioning, fatigue, summary (preference-based utility scores) and domain-specific QoL (urinary incontinence, bowel function and erectile function), cancer specific distress, psychological distress, decision-related distress and time to return to usual activities. Outcomes are being measured pre-operatively and at 6 weeks and 3, 6, 9, 12, 18 and 24 months post-surgery. Co-morbidity is being considered as a critical issue in deciding who may ultimately benefit from treatment. An economic evaluation is addressing the question of whether open or robotic RP is better ‘value-for-money’, having regard to their comparative costs and outcomes [12-14]. The economic model is integrating the surgical/clinical indices and QoL data from the trial, with the resource costs of the inputs necessary to implement both intervention arms.

## Methods/design

### Study aims and hypotheses

The study aims are to assess the quality of life effects of robotic surgery compared with open prostatectomy; determine the economic costs of robotic versus open prostatectomy with regards to both health sector costs and direct and indirect cost to patients; evaluate life expectancy profiles of patients receiving both forms of prostatectomy.

We hypothesise that:

1. There will be no difference between the two groups in terms of surgical and clinical indices of oncological outcome.

2. Patients randomised to robotic prostatectomy will have improved short term (3 months postoperative) quality of life outcomes compared with those randomised to open prostatectomy with regards to pain, physical functioning, fatigue; summary (AQoL-8D and SF36) and domain specific quality of life (urinary incontinence, bowel function and erectile function), time to return to usual activities.

3. Patients randomised to open prostatectomy will have comparable medium (6–12 months) and long term (24 months) quality of life outcomes compared with those randomised to robotic prostatectomy with regards to pain, physical functioning, fatigue; summary (AQoL-8D and SF36) and domain specific quality of life (urinary incontinence, bowel function and erectile function).

4. There will be no difference between the two groups with regards to psychological distress, cancer specific distress and decision-related distress.

5. There will be a short-term and overall cost advantage for open prostatectomy.

Men are being recruited via the Royal Brisbane and Women’s Hospital (RBWH) Urology Outpatient Clinic and urologists’ private practices. Inclusion criteria are that the men must: (1) be newly diagnosed with localised PCa and have chosen surgery as their treatment approach (2) be able to read and speak English (3) have no previous history of head injury, dementia or psychiatric illness (4) have no other concurrent cancer. Following a pilot study of 69 patients earlier in 2010, recruitment commenced in October of that year and is continuing to allow for a total sample size of 400 men randomly allocated to either robotic RP or open RP. Since commencement of the definitive trial, we have recruited and randomised 146 men, 141 of whom have had a radical prostatectomy (73 = robotic; 68 = open). Five men randomised to open prostatectomy did not proceed in the trial; two elected to have a robotic prostatectomy privately, two had life-threatening cardiac problems following induction of anaesthesia such that the operation was aborted and one had previous laparoscopic inguinal hernia repairs using mesh so that access to the prostate was severely compromised. All three proceeded to have external beam radiation therapy, following neo-adjunctive androgen deprivation therapy, with curative intent. Patients are assessed at recruitment and before treatment (baseline), then again at 6 weeks and 3, 6, 12 and 24 months post-surgery.

### Study integrity

Ethical approval has been obtained from the RBWH Human Research Ethics Committee. The study design is guided by the CONSORT statement [15]. All men provide written informed consent. Assessments are by self-report pen and paper measures with project staff tracking assessments. Randomisation occurs in blocks of 10, with each condition randomly generated 5 times within each block to ensure an unpredictable allocation sequence with equal numbers of men in each group at the completion of each block. This sequence is undertaken using a computerised database independent from the clinical team and research staff. Stratification occurs by age group (40–49 yrs, 50–59 yrs, 60-69 yrs); and IPSS score (0–7, 8–19, 20–35). Following randomisation, patients are telephoned the same day to be notified of their designated surgical procedure and posted an information booklet outlining expectations for their scheduled operation. Following this, patients are placed on the surgical waiting list at the RBWH and scheduled for surgery as per standard hospital procedures. Biopsy slides are reviewed pre-operatively by staff pathologists JP-K, JDP or MLTHS all of whom have recognised expertise in uropathology.

## Materials

QoL and other outcome assessments are being undertaken using a series of well-validated and reliable measures administered by pen and paper questionnaire. Socio-demographic information is being collected at time of recruitment by interview. Clinical diagnostic and treatment information, including operative time, length of hospital stay, blood loss, time to catheter removal, use of narcotic analgesia and peri-operative complications are being collected through medical record review. Pathology review is being undertaken centrally by our two uropathologists to the trial in conjunction with MLTHS and includes standardised evaluation of the Gleason scoring using the 2005 ISUP classification, positive margins, extra prostatic capsular extension, neurovascular and seminal vesicle involvement with quality control of selected sections by international pathologists. Prostate-specific antigen testing occurs 2–4 weeks pre-operatively; then at 6 weeks, 3, 6, 9, 12, 18 and 24 months after surgery; and then annually. Biochemical failure (bF) is diagnosed on the first occasion following prostatectomy that the serum PSA is ≥0.2 ng/mL, after which patients undergo total body bone scan and CT scan of abdomen and pelvis for assessment of local and distant failure. Patients are given adjuvant or salvage radiation therapy at the surgeon’s discretion. Clinical staging is being performed using the current UICC TNM staging classification based on the outcome of biopsies, digital rectal exam, transrectal ultrasonography (TRUS), bone scan and contrast-enhanced computerised tomography (CT) pre-operatively. MRI is performed at the surgeon’s discretion. A CT scan of the pelvis and abdomen with 8 mm-slices is performed to evaluate lymph nodes. Pathological staging is being determined from histopathology of the radical prostatectomy specimen along with the findings from lymph nodes with extended lymph node dissection performed for patients with serum PSA >10 ng/ml, primary Gleason ≥4 or cT2b or greater. Postoperative complications are being assessed through chart review supplemented by discussion and letter sent with the patient questionnaire according to the Clavien system of classification.

### Self-report measures

#### Anxiety and depression

The Hospital Anxiety and Depression Scale (HADS; [16]) is providing a measure of current psychological distress with subscale scores for anxiety and depression.

### Psychological distress

The Revised Impact of Events Scale (IES-R [17]) is being used to measure men’s cancer specific distress symptoms. The IES-R has three subscales: intrusion, avoidance and hyperarousal. Internal consistencies for the IES-R subscales are good. Epping-Jordan [18] suggest that intrusion and avoidance are more sensitive measures of psychological distress after a cancer diagnosis than generalised distress measures. In men treated for localised prostate cancer intrusion and avoidance have been found to be related to poorer mental health outcomes [19].

### Decision-related distress

For decision-related distress we assess both decisional conflict and decision regret. The Decisional Conflict Scale-Revised (DCS [20,21]) measures a person’s perception of the difficulty involved in making a decision about medical treatments. The revised scale has 19 items covering decisional uncertainty, feeling uninformed, unclear about personal values, and unsupported in decision making, and perceptions of effective decision making. The DCS has been validated in a range of population groups and is sensitive to people making different health decisions, and to the effect of decision aids, with good internal consistency for the total scale. Health decisions assessed with this scale include treatment for localised prostate cancer.

### Domain specific QoL

Domain specific QoL is being measured by the EPIC, the Expanded Prostate Cancer Index Composite [22]: 39 items from this measure will assess functioning in three problem domains: urinary, sexual and bowel. Men are also completing the International Index of Erectile Functioning (IIEF) [23], which allows sexual function to be assessed in five domains: erectile function, orgasmic function, sexual desire, intercourse satisfaction and overall sexual satisfaction. A scale developed by Schover [24] is being used to assess whether men have obtained medical help for sexual dysfunction and the impact of each treatment on their sex life.

### Health related quality of life

Health related quality of life is being assessed with the SF-36 that is the most widely used QOL measure in the world with norms for the Australian general population available. The SF-36 [25] contains a mental health and physical health summary scale suitable to measure the impact of the intervention on patients’ wellbeing. As well, the Assessment of Quality of Life – 8D (AQoL-8D) will be administered [26]. The AQoL-8D is a 35-item scale assessing quality of life on eight dimensions including: independent living, relationships, mental health, self worth, happiness, coping, pain, and sensory perception. The AQoL-8D is the primary outcome instrument for the economic appraisal, has excellent psychometric properties and has been used in over 80 trials in Australia.

### Employment outcomes

Questions are being asked on pre-cancer and current employment status, time off work, returning to work, receipt of sickness and other benefits, unpaid work activities, changes in work role, consequences at work due to cancer, perceived work productivity, assistance from family or others, and carer’s or partner’s work patterns since the participant’s cancer diagnosis.

### Prospective life expectancy modelling

A secondary analysis will identify the comorbidity index which optimises prediction of death from causes other than prostate cancer within 10 years of diagnosis of PCa. We will use the ChI, age-adjusted ChI, ASA, number of comorbid conditions, number of prescription medications (data collected as part of routine history and examination on a proforma) and the TIBI-CaP (an 84 question questionnaire for the patient) as predictor covariates. Time to death from comorbid conditions, as well as from PCa, will be assessed at 5 and 10 years following trial closure. The discriminatory ability of various indices will be assessed using concordance index of the covariate (analogous to area under the ROC curve), with statistical differences between models tested using Integrated Discrimination Index (IDI).

### Economic appraisal

Costs are being assessed from a health sector perspective, covering impacts that fall on ‘government as 3rd party funder’, as well as those affecting patients and their families. Pathway analysis (incorporating decision tree analysis) will be used to clearly identify the activity components for each arm of the trial, complemented by a prospective patient-held cost diary covering the entire trial period (week one to 24 months). The cost categories in the diary are those of travel costs, time costs and productivity costs. Patients fill in the cost diary at one week and then are discharged home with the diary to fill out at home. At each visit patients return the completed cost diary and collect the cost diaries relevant to the next period of the trial.

Costs for each arm of the trial will be analysed by components of the intervention pathway (e.g. pre-consultation, work-up, theatre, post-treatment, follow-up, management/coordination, side-effects, etc.); by expenditure category (e.g. capital, staff, consumables, overheads, other); and by incidence (i.e. who bears the costs). The cost component of the economic appraisal will thus assess the cost drivers from a range of perspectives for both intervention approaches. Downstream costs and potential cost offsets incurred beyond the randomised trial data collection period will be modelled from the literature and expert opinion, with sources clearly documented. Production gains/losses in the general economy will be modelled based on data collections from the trial, using methods developed by Deakin Health Economics [27,28]. Cost-effectiveness results will be reported with and without production effects included.

The economic appraisal will involve a cost effectiveness analysis (CEA), a cost consequences analysis (CSA) and a cost utility analysis (CUA). Outcome measures in the CSA will include incremental changes in the full range of clinical/surgical and psychosocial variables, to paint as comprehensive a picture as possible between resource usage and outcomes. In the CEA outcomes will be reported as incremental cost per outcome ratios (ICERs) for a select range of outcomes that have clinical and/or policy relevance. ICERs will be reported for return to work, the EPIC (the 3 domains urinary, sexual and bowel) and the IIEF (the 5 domains of sexual function). In the CUA, outcomes will be reported as the ‘incremental cost per QALY’ (quality adjusted life year). The AQol-8D will be used as the primary measure of QoL in the economic appraisal, with secondary ICERs developed for the SF-36. Consistency between the QoL results from the AQoL-8D, the SF-36 and the other domain specific QoL measures collected in the trial will be carefully assessed. Standard discounting will be applied to both costs and outcomes, together with detailed sensitivity testing.

### Statistical analyses

The study design involves a two group Randomised trial with five assessment points. As the hypotheses involve comparisons of the rate of change with which men in the treatment versus control groups will achieve optimal physical and psychological QoL, the appropriate analysis is a two level multilevel model (with Level 2 = men and Level 1 = Assessment points). These analyses will be adjusted to account for equivalence testing procedures where appropriate with respect to the hypotheses. Although power models are not articulated for multilevel analysis as clearly as they are for ordinary linear modelling, the Optimal Design software of Raudenbush [29] and the simulation work of Jo [30] suggest appropriate sample sizes. Assuming that at least 70% of the men in each group will remain in the study (this is highly conservative as we have previously demonstrated only a 10% attrition rate in QoL studies with men with PCa) and assuming further moderate effect size of d = 0.5 and alpha at .05, a total sample size if 400 (i.e. 200 per group) would result in over 90% power.

## Discussion

This research program is addressing key questions of importance by evaluating robotic and open prostatectomy in terms of oncologic parameters, quality of life effects including level of satisfaction, economic costs and overall patient life expectancy; and more broadly by establishing a gold standard for the evaluation of new medical technologies in the clinical treatment of prostate cancer in Australia and internationally. Outputs include evidence to facilitate more effective decision making about surgical treatment for localised prostate cancer and information to support health service planning and development for this rapidly growing patient group.

## Competing interests

The authors declare that they have no competing interests.

## Authors’ contributions

RAG developed the study concept and aims and initiated the project. All authors assisted in further development of the protocol. RAG and SKC were responsible for drafting the manuscript. RAG, JY, GC, ND, RC implement the protocol and oversee collection of the data. All authors contributed to the final manuscript.

## Pre-publication history

The pre-publication history for this paper can be accessed here:

http://www.biomedcentral.com/1471-2407/12/189/prepub
